# Prognostic value of 18F-FDG PET /CT metabolic parameters in patients with locally advanced pancreatic Cancer treated with stereotactic body radiation therapy

**DOI:** 10.1186/s40644-020-00301-6

**Published:** 2020-03-10

**Authors:** Shengnan Ren, Xiaofei Zhu, Anyu Zhang, Danni Li, Changjing Zuo, Huojun Zhang

**Affiliations:** 1 Department of Nuclear Medicine, Shanghai Changhai Hospital, No. 168 Changhai Road, Shanghai, 200433 China; 2 Department of Radiation Oncology, Shanghai Changhai Hospital, No. 168 Changhai Road, Shanghai, 200433 China

**Keywords:** Locally advanced pancreatic cancer, Prognosis, ^18^F-FDG PET/CT, Stereotactic body radiotherapy, Cyberknife radiosurgery

## Abstract

**Background:**

^18^F-FDG PET/CT metabolic parameters have been applied as prognostic factors in multi-malignancies. However, the role in locally advanced pancreatic cancer (LAPC) was not confirmed. In this study, we investigated the prognostic value of ^18^F-FDG PET/CT metabolic parameters in LAPC patients treated with stereotactic body radiation therapy (SBRT).

**Methods:**

Seventy three LAPC patients who received SBRT therapy and pre-treatment ^18^F-FDG PET/CT imaging from January 2012 to January 2016 were included in this retrospective study. The study aim was to evaluate the relationship between metabolic parameters with clinical factors, and the value of metabolic parameters in the prognosis of LAPC. The median of parameters was set as the cut-off value for statistical analysis. Univariate survival analysis was performed by the Kaplan Meier method and log-rank test, and multivariate analysis was carried out by a Cox proportional hazards model.

**Results:**

Patients with lymph node metastasis or longer tumor diameters were associated with higher TLG (*P* < 0.05). Univariate analysis showed MTV, TLG, radiotherapy dose and chemotherapy were significantly associated with disease progression-free survival (PFS) and overall survival (OS) (*P* < 0.05). Lymph node metastasis and tumor longest diameter were associated with OS. Multivariate analysis demonstrated TLG, radiotherapy dose, and chemotherapy were independent factors of PFS and OS (HR: 2.307, 0.591, 0.572 and 2.145, 0.480, 0.471, *P* < 0.05).

**Conclusions:**

TLG was found to be the independent prognostic factor of OS and PFS. Among clinical factors, radiotherapy dose and chemotherapy were independent prognostic factors of OS and PFS.

## Background

Pancreatic cancer is one of the malignancies with extremely poor prognosis. Its one-year survival rate is about 15%, and less than 5% of pancreatic patients survived more than 5 years [1]. Currently, surgery remains to be the only cure for pancreatic cancer. However, because of the hidden location, and lack of typical symptoms in the early stages, only about 20% of patients showed surgical indications at the time of diagnosis. About 20–40% of patients are diagnosed with locally advanced pancreatic cancers (LAPC), whose median survival period was 6–14 months. Of the 40–45% of pancreatic cancer patients who present with distant metastases, the median survival time was only 3–6 months [2].

According to the American Society of Clinical Oncology (ASCO) guidelines, the recommended therapies for LAPC includes mono-chemotherapy, multi-agent chemotherapy, and chemoradiotherapy (CRT) [3]. Cyberknife, a newly developed Stereotactic body radiation therapy (SBRT) device, has been widely used in cancer treatment. The new technology is a precise radiotherapy mode guided by real-time imaging, which means a higher dose of radiation in the target areas and fewer side effects. Patients who received SBRT barely showed serious adverse reactions in relevant studies [4]. Moreover, 86% of SBRT treated patients illustrated obvious relief in pain, and 78% of which showed disease-controlling effect. In phase III clinical trial [5], SBRT was described as a potential standard treatment for pancreatic cancer under certain circumstances.

Most LAPC patients died of local recurrence or post-treatment metastasis. The low survival rate and non-durable treatment responses make early-stage prognostic evaluation particularly important for LAPC patients. ^18^F-FDG PET/CT is a combination of functional imaging and anatomical imaging, which has been clinically applied in diagnosis, staging, recurrence, and treatment efficacy evaluation of multiple malignancies [6, 7]. Research performed by Schellenberg et al. [8] showed that the pre-treatment SUV_max_ was associated with the overall survival (OS) and progression-free survival (PFS) of pancreatic cancer patients. In recent studies, another two ^18^F-FDG PET/CT volumetric metabolic parameters, tumor metabolic volume (MTV) and total lesion glycolysis (TLG), showed greater value in the evaluation of pancreatic cancer prognosis than SUV_max_ [9, 10]. However, the limited numbers of the current studies about ^18^F-FDG PET/CT parameters in LAPC prognosis failed to show any consistent result. Moreover, studies on ^18^F-FDG PET/CT metabolic parameters in prognostic value of LAPC treated with SBRT by Cyberknife are rare. In our previous study [11], ^18^F-FDG PET/CT metabolic parameters were tested for the prognostic value among 23 LAPC patients who received chemotherapy as well as SBRT. In this study, besides the patients received both chemotherapy and SBRT, we expanded the criteria to include the LAPC patients treated with SBRT only. In order to further discuss the individual-based prognosis assessment for LAPC patients.

## Methods

All methods included in this study were carried out in accordance with relevant guidelines and regulations. All experimental protocols were approved by the Institutional Review Board of Changhai Hospital.

### Patients

We retrospectively analyzed patients who underwent ^18^F-FDG PET/CT scan and received SBRT at our institution between January 2012 to January 2016. Informed consent was obtained from all subjects.

#### Inclusion criteria

Firstly, pathologically confirmed as pancreatic cancer. Secondly, Underwent ^18^F-FDG PET/CT scan within 2 weeks before SBRT treatment.

#### Exclusion criteria

Firstly, distant metastasis found by ^18^F-FDG PET/CT. Secondly, presence of cocurrent malignancies other than pancreatic cancer. Thirdly, previous history of anti-tumor treatment at the pancreas site. Fourthly, Deaths caused by diseases other than pancreatic cancer during follow-ups. Lastly, patients who were not willing to obey the treatment plan or examination schedule during follow-ups.

In the end, medical data of 73 patients was collected and retrospectively analyzed.

### ^18^F-FDG PET/CT

All scans were performed before treatment, patients were injected with ^18^F-FDG (radiochemical purity ≥95%, Shanghai Atomic Kexing Pharmaceutical Co., Ltd.,) at a dose of 3.70–5.55 MBq/kg. At least 6 h of fasting was applied to all patients prior to scanning, and the blood glucose level was reported below 11.1 mmol/L. All acquisition was performed on a Biograph tripoint 64-layer 52-ring HD PET/CT scanner (Siemens, Germany), 45–60 min after the injection of ^18^F-FDG. PET images were performed on 5–6 bed positions with 2.5 min per bed position from mid-skull to mid-thigh. As for CT scanning parameters, slice thickness was 3 mm, acquisition time was 18.67 to 21.93 s, under 170 mA and 120 kV voltage.

### Analysis of ^18^F-FDG PET/CT images

The three-dimensional and fusion images were reconstructed by using the ordered subset expectation maximization (OSEM) and the Multimodality multi-mode workstation, respectively. Two experienced nuclear medicine physicians, who were both blinded to the outcomes, reviewed all images. And spherical regions of interest were circled around the identified lesion tissues afterward. SUV_max_, SUV_mean_, MTV, and TLG were offered by the TrueD system automatically. The ceCT or MRI images were used to help differentiate inflammatory activity from tumor tissue if necessary. MTV was evaluated at the baseline of 40% of SUV_max_. The longest tumor diameter was measured on CT images. Meanwhile, the lymph nodes with a short axis diameter greater than 1 cm were considered as lymph node metastases.

### Therapies

#### Stereotactic body radiation therapy delivery

Radiation therapies were carried out by the Cyberknife® (Accuray Incorporated, Sunnyvale, United States)_,_ an image-guided frameless treatment system. The vertebral tracking technology was used to provide real-time imaging, which replaced the motion detection by gold fiducials implantation. All patients underwent a ceCT or MRI scan before radiation therapy for treatment boundary assessment. RADIOTHERAPY was defined as the CT evidenced total tumor range. The planning target volume (PTV) included the whole radiotherapy area with extended margins (2 to 3 mm) in X, Y, and Z axis. The routine dose for single irradiation was 6–8 Gy, 4 to 8 repeats for a course. And the treatment parameters were adjustable between different individual situations.

### Chemotherapy devilry

Fifty LAPC patients received single SBRT treatment because of the chemotherapy intolerance. 23 patients received chemotherapy and SBRT combination therapy. Among which, 13 patients received gemcitabine-based chemotherapy, 9 patients received S-1 based chemotherapy, and 1 patient received gemcitabine -S-1 multi-agent chemotherapy.

### Follow-ups and statistical analysis

Patients were followed up by telephone or through the clinic. OS and PFS were used as prognostic indicators. OS refers to the time duration between the day therapy begun to the day of disease-related death or last visit. PFS was defined as the period from the completion of treatment to disease progression, including local progression distant metastasis, or any causes of death or the last follow-up in the case of no progression. Disease progression was evaluated by RECIST criteria (v1.1). Patients included were required to take enhanced contrast CT and MRI every 2 to 3 months during follow-up.

Statistical analysis was performed using SPSS version 22 statistical software (Portsmouth, UK). Mann-Whitney U or Kruskal-Wallis H test was applied for the correlation between clinical factors and metabolic parameters. All continuous variables were presented as median value M (P25, P75). For survival analysis, patients were put into two groups (high-value group and low-value group) based on the medium value of continuous variables. Afterward, all data were analyzed by the Kaplan-Meier methods for the purpose of univariate analysis, and then significant variables were included in the Multivariate step-wise forward Wald–Cox regression. *P* value < 0.05 was considered as statistically significant.

## Results

### Relationship between clinical factors and metabolic parameters

The median values of SUVmax, SUVmean, MTV and TLG in 73 LAPC patients were 6.9 (P25: 5.85, P75: 9.85), 4.0 (P25: 3.2, P75: 5.6), 12.2 (P25: 7.0, P75: 22.5) cm^3^, and 49.3 (P25: 31.6, P75: 86.0) g, respectively. SUVmax, SUVmean and TLG of patients with lymph node metastasis were 8.6 (P25: 5.9, P75: 11.2), 4.9 (P25: 3.3, P75: 5.7), and 67.8 (P25: 37.9, P75: 117.1) g, respectively, which were significantly higher than those without lymph node metastasis (6.7 (P25: 5.6, P75: 8.1), 3.7 (P25: 3.1, P75: 4.4), and 45.6 (P25: 27.9, P75: 64.4) g, respectively (*P* = 0.029, 0.048 and 0.007, respectively). Moreover, a longer tumor diameter was associated with higher TLG (*P* = 0.035). No significant differences were found between MTV and clinical factors (all *P* > 0.05, Table 1).

**Table 1:** Correlation between Clinical Factors and SUVmax, SUVmean, MTV and TLG in 73 LAPC Patients

Clinical Factors	Cases	SUVmax			SUVmean		
		M (P25, P75)	z Value or χ2 Value	*P* Value	M (P25, P75)	z Value or χ2 Value	*P* Value
Age (yrs)			− 0.387	0.699		−0.392	0.695
≤ 68	38	7.0 (6.1, 9.7)			4.1 (3.4, 5.7)		
>68	35	6.6 (5.3, 10.1)			3.8 (3.0, 5.5)		
Presence of DM			−0.533	0.594		−0.232	0.816
No	58	6.9 (5.9, 10.0)			4.0 (3.2, 5.7)		
Yes	15	6.8 (5.8, 8.5)			4.0 (3.0, 5.3)		
ECOG			0.838^a^	0.658		1.742^a^	0.418
0	13	6.7 (6.4, 9.9)			4.0 (3.5, 5.4)		
1	32	7.1 (5.5, 11.3)			4.3 (2.9, 6.7)		
2	28	6.6 (5.2, 9.1)			3.7 (3.0, 5.2)		
N Stage			−2.184	0.029		−1.974	0.048
0	40	6.7 (5.6, 8.1)			3.7 (3.1, 4.4)		
1	33	8.6 (5.9, 11.2)			4.9 (3.3, 6.5)		
Position			−0.709	0.478		−0.746	0.456
Head	51	6.8 (6.0, 10.2)			4.1 (3.4, 5.5)		
Body/ Tail	22	7.0 (5.28, 9.6)			3.8 (3.0, 5.7)		
The longest diameter (cm)			−1.353	0.176		−1.054	0.292
≤ 3.7	40	6.7 (5.8, 8.4)			3.9 (3.1, 5.1)		
> 3.7	33	7.3 (6.0, 11.1)			4.2 (3.1, 6.6)		
CA 19–9 (ng/mL)			−0.590	0.555		−0.497	0.619
≤ 321.6	37	6.7 (5.7, 9.6)			4.0 (3.0, 5.4)		
> 321.6	36	7.1 (5.8, 10.0)			4.1 (3.3, 5.7)		
Clinical Factors	Cases	MTV (cm^3^)			TLG (g)		
		M (P25, P75)	z Value or χ2 Value	*P* Value	M (P25, P75)	z Value or χ2 Value	P Value
Age (yrs)			−0.961	0.337		−0.939	0.348
≤ 68	38	11.3 (6.5, 19.8)			48.2 (29.6, 75.4)		
>68	35	13.2 (7.3, 27.1)			58.1 (33.9, 98.4)		
Presence of DM			−0.608	0.543		0.000	1.000
No	58	11.8 (7.3, 20.0)			49.0 (34.2, 85.2)		
Yes	15	15.9 (5.2, 35.5)			62.2 (25.4, 146.3)		
ECOG			0.331^b^	0.847		0.063^b^	0.969
0	13	12.2 (7.0, 20.5)			48.7 (32.3, 83.5)		
1	32	10.5 (5.6, 23.7)			51.0 (29.9, 88.9)		
2	28	12.6 (9.2, 25.9)			49.4 (34.0, 93.9)		
N Stage			−1.336	0.182		−2.682	0.007
0	40	12.1 (6.3, 15.7)			45.6 (27.9, 64.4)		
1	33	13.9 (7.0, 35.6)			67.8 (37.9, 117.1)		
Position			−1.455	0.146		−1.353	0.176
Head	51	11.7 (6.7, 5.9)			46.8 (28.6, 82.1)		
Body/ Tail	22	20.2 (7.8, 28.5)			61.1 (39.7, 99.1)		
The longest diameter (cm)			−1.596	0.110		−2.111	0.035
≤ 3.7	40	10.0 (5.6, 18.9)			41.7 (26.0, 77.5)		
> 3.7	33	13.9 (8.4, 24.2)			64.7 (40.0, 91.3)		
CA 19–9 (ng/mL)			−0.083	0.934		−0.535	0.593
≤ 321.6	37	12.6 (7.5, 17.9)			48.7 (32.2, 80.6)		
> 321.6	36	10.5 (6.4, 25.2)			55.4 (30.7, 92.5)		

*LAPC* locally advanced pancreatic cancer; *SUV* standardized uptake value; *MTV* metabolic tumor volume; *TLG* total lesion glycolysis; *DM* diabetes mellitus; *ECOG* Eastern Cooperative Oncology Group

a, b: χ2 Value

*: *P* < 0.05

### Patient characteristics

Seventy three LAPC patients (46 males, 27 females) from January 2012 to January 2016 were enrolled in this study, the basic characteristics and pre-treatment conditions were summarized in Table 2. The median follow-up duration was 37 months (range: 12–55 months). For OS, there were 6 cases with censored data, including 2 patients lost follow-ups and 4 patients who were alive. And the rate of lost follow-ups was 2.7%. The median PFS time was 9.7 months (95% CI: 9.1–10.3), only one case presented with censored data in PFS, who showed no evidence of disease progression by the end of follow-up. The 1 year and 2 years PFS rate was 30.1 and 4.1%, respectively. Meanwhile, the median OS period was 14.4 months (95% CI: 13.3–15.5). The 1-year and 2-year OS rates were 78.1 and 11.2%, respectively.

**Table 2:** Patient characteristics

Characteristics	Total (*n* = 73)
Gender	
Male	46
Female	27
Age (yrs), Median (range)	68 (42–84)
N Stage	
N_0_	40
N_1_	33
Tumor Location	
Head	51
Body/Tail	22
Longest Diameter of Tumor (cm), Median (range)	3.7 (1.0–7.5)
Serum CA19–9 Level (ng/mL), Median (range)	321.6 (2.0–1200.0)
Present of DM	15
ECOG Score	
0	13
1	32
2	28

*DM* diabetes mellitus; *ECOG* Eastern Cooperative Oncology Group

### Survival analysis and univariate analysis

**Fig. 1:**
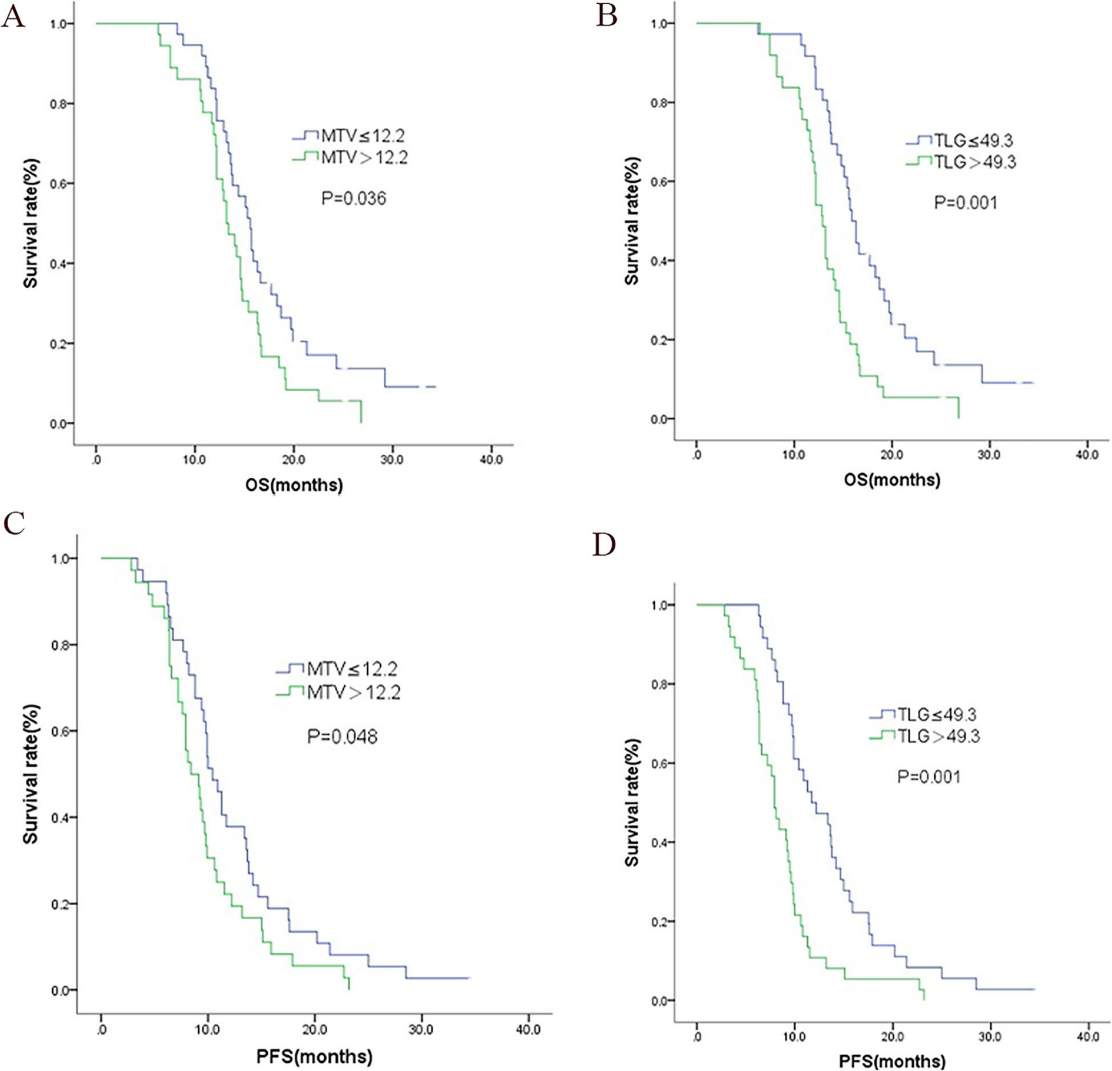
Kaplan-Meier univariate analysis of OS according to MTV (**a**) and TLG (**b**), PFS according to MTV(**c**) and TLG (**d**)

The log-rank test was used to analyze metabolic parameters, including MTV and TLG (Fig. 1, Table 3), and we found MTV and TLG were prognostic factors of OS and PFS. The median overall survival time was 15.6 (95% CI: 14.1–17.2) months and 13.2 (95% CI: 11.6–14.8) months for patients with MTV ≤ 12.2 and MTV > 12.2 respectively (*P* = 0.036, HR:1.669,95% CI: 1.025–2.716). The median overall survival time was 15.9 (95% CI: 14.9–16.9) months for patients with TLG ≤ 49.3, and 12.9 (95% CI: 12.1–13.7) months for patients with TLG > 49.3, respectively (*P* = 0.001, HR: 2.320,95% CI: 1.413–3.809).

**Table 3:** Univariate Analysis of Prognostic Factors for 73 LAPC patients

Factors	Cases	OS					PFS				
		Cases Of Death	Median OS (Month)	95% CI	χ2 Value	*P* Value	Cases of Progression	Median PFS (Month)	95% CI	χ2 Value	*P* Value
Gender					0.010	0.918				0.342	0.559
Male	46	44	14.6	12.9–16.3			45	9.8	9.1–10.5		
Female	27	23	13.8	12.1–15.5			27	9.4	8.0–10.8		
Age (yrs)					2.284	0.131				2.041	0.153
≤ 68	38	34	15.3	13.8–16.8			37	9.4	8.2–10.6		
>68	35	33	13.6	12.1–15.1			35	10.0	8.3–11.7		
Presence of DM					0.308	0.579				0.683	0.409
No	58	54	13.8	12.1–15.5			57	9.6	8.7–10.5		
Yes	15	13	14.6	13.7–15.5			15	10.8	8.0–13.6		
ECOG					0.485	0.785				0.006	0.997
0	13	12	13.2	9.8–16.6			12	9.8	7.1–12.5		
1	32	30	13.8	13.0–14.6			32	9.6	8.8–10.4		
2	28	25	14.7	13.0–16.4			28	9.4	8.4–10.3		
N Stage					4.748	0.029*				2.267	0.132
0	40	35	15.1	13.1–17.1			40	9.9	6.8–13.0		
1	33	32	13.2	12.2–14.2			32	9.2	7.9–10.6		
Tumor Location					0.029	0.864				0.000	0.990
Head	51	46	14.6	13.1–16.1			50	9.8	9.3–10.3		
Body/Tail	22	21	14.0	12.4–15.6			22	8.8	7.3–10.3		
Longest Diameter (cm)					4.010	0.045*				2.196	0.138
≤ 3.7	40	35	14.8	13.7–15.9			40	10.0	8.6–11.4		
>3.7	33	32	13.2	11.9–14.6			32	9.1	7.9–10.3		
CA19–9 (ng/mL)					3.391	0.066				3.126	0.077
≤ 321.6	37	32	15.3	14.1–16.5			36	10.4	9.1–11.7		
>321.6	38	35	13.2	11.9–14.5			36	8.8	7.2–10.4		
Radiotherapy Dose (Gy)					13.907	<0.001*				9.834	0.002*
≤ 37.2	37	34	12.2	11.3–13.2			36	7.9	7.0–8.9		
>37.2	36	33	16.6	15.4–17.8			36	11.3	7.8–14.8		
Chemotherapy					7.603	0.006*				4.690	0.030*
No	50	49	13.8	12.7–15.0			49	9.5	8.5–10.5		
Yes	23	18	16.7	11.5–21.9			23	11.3	4.9–17.7		
SUV_max_					0.747	0.387				0.205	0.651
≤ 6.9	38	34	14.7	13.3–16.1			38	9.8	8.9–10.7		
>6.9	35	33	13.7	12.3–15.1			34	9.5	8.3–10.7		
SUV_mean_					0.310	0.578				0.074	0.785
≤ 4.0	38	34	14.7	13.3–16.1			38	9.8	8.9–10.7		
>4.0	35	33	13.7	12.3–15.1			34	9.5	8.3–10.7		
MTV (cm^3^)					4.401	0.036*				3.913	0.048*
≤ 12.2	37	32	15.6	14.1–17.2			36	10.4	9.1–11.7		
>12.2	36	35	13.2	11.6–14.8			36	8.4	6.5–10.3		
TLG (g)					11.843	0.001*				13.777	<0.001*
≤ 49.3	36	31	15.9	14.9–16.9			35	11.7	8.0–15.4		
>49.3	37	36	12.9	12.1–13.7			37	7.9	7.0–8.9		

*LAPC* locally advanced pancreatic cancer; *OS* overall survival; *PFS* progression-free survival; *DM* diabetes mellitus; *ECOG* Eastern Cooperative Oncology Group; *SUV* standard uptake value; *MTV* metabolic tumour volume; *TLG* total lesion glycolysis

*: *P* < 0.05

The median PFS time of patients with MTV ≤ 12.2 and MTV > 12.2 was 10.4 (95% CI:9.1–11.7)months and 8.4(95% CI:6.5–10.3)months (*P* = 0.048, HR: 1.601, 95% CI: 0.998–2.567), respectively. The median PFS time of patients with TLG ≤ 49.3 and TLG > 49.3 was 11.7(95% CI:8.0–15.4) months and 7.9(95% CI: 7.0–8.9) months, respectively (P = 0.001, HR: 2.424 1,95% CI: 1.495–3.939).

As for clinical factors, Lymph node metastasis, longest diameter of tumor, radiotherapy dose and chemotherapy were significant OS-related prognostic factors (*P* = 0.029, 0.045, 0.001 and 0.006, respectively). Patients with lymph node metastases, longer tumor diameters or lower radiotherapy dose tended to have shorter OS. Meanwhile, LAPC patients treated without chemotherapy also showed a shorter OS duration. In addition, radiotherapy dose and chemotherapy were significantly associated with PFS (*P* = 0.002 and 0.030, respectively). LAPC patients received higher radiotherapy dose or combined with chemotherapy demonstrated a longer PFS time (Table 3).

### Multivariate analysis of prognostic factor

Multivariate analysis performed by Cox proportional hazards models showed that TLG, radiotherapy dose, and chemotherapy were independent prognostic factors for OS and PFS.

The hazard ratio (HR) of TLG, radiotherapy dose and chemotherapy upon OS was 2.145 (*P* = 0.003, 95% CI: 1.292–3.560), 0.480 (*P* = 0.004, 95% CI: 0.289–0.796) and 0.471 (*P* = 0.010, 95% CI: 0.267–0.833), respectively. The HR of TLG, radiotherapy dose and chemotherapy upon PFS was 2.307 (*P* = 0.001, 95% CI: 1.406–3.787), 0.591 (*P* = 0.033, 95% CI: 0.364–0.960) and 0.572 (*P* = 0.040, 95% CI: 0.335–0.976), respectively. All *P* < 0.05 (Table 4).

**Table 4:** Multivariate Analysis of Prognostic Factors for OS and PFS in 73 LAPC patients

Factors	OS					PFS			
	B Value	Wald Value	*P* Value	HR (95%CI)		B Value	Wald Value	*P* Value	HR(95%CI)
Radiotherapy dose	−0.735	8.090	0.004	0.480(0.289~0.796)		−0.525	4.521	0.033	0.591(0.364~0.960
Chemotherapy	−0.752	6.698	0.010	0.471(0.267~0.833)		− 0.559	4.198	0.040	0.572(0.335~0.976)
TLG	0.763	8.704	0.003	2.145(1.292~3.560)		0.836	10.938	0.001	2.307(1.406~3.787)

*LAPC* locally advanced pancreatic cancer; *OS* overall survival; *PFS* progression-free survival; *TLG* total lesion glycolysis

Among clinical factors, radiotherapy dose and chemotherapy were founed as indepent prognostic factors. Mann-Whitney U test was used verify the distribution difference of clinical factors between two groups of patients with or without chemotherapy, and between two groups with high or low radiotherapy dose (Table 5). The statistical results indicated that there was no statistical difference in the distribution of clinical factors between the chemotherapy group (23 cases) and the non-chemotherapy group (50 cases). The negative results also showed between the low radiotherapy dose group (37 cases) and the high radiotherapy dose group (36 cases). According to statistical data, the selection bias possibly caused by retrospective study could be reduced.

**Table 5:** Distribution of Clinical Factors upon with/without Chemotherapy and high/low radiotherapy dose

Clinical Factors	Chemotherapy		Radiotherapy Dose	
	z Value	P Value	z Value	P Value
Age, Median	− 0.515	0.607	− 1.517	0.129
Gender	−1.299	0.194	−0.633	0.527
N Stage	−1.205	0.228	−1.062	0.288
Serum CA19–9, Median	−0.171	0.864	−0.115	0.909
Present of DM	−0.789	0.430	−1.498	0.134
Longest Diameter, Median	−1.205	0.228	−1.062	0.288
Tumor Location	−0.037	0.970	−1.596	0.110
ECOG Score	−0.096	0.923	−1.881	0.060

*DM* diabetes mellitus; *ECOG* Eastern Cooperative Oncology Group

**Fig. 2:**
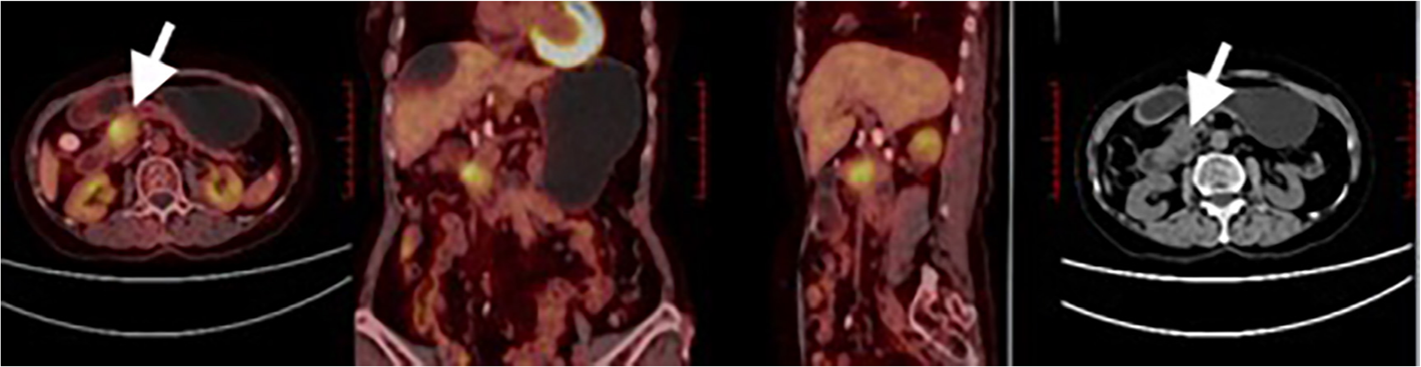
Female, 73 years old, pancreatic head cancer. ^18^F-FDG PET/CT fusion images showed a mass in the pancreatic head, with increased radioactivity uptake. SUV_max_ 6.3, SUV_mean_ 3.6, MTV 5.4 cm^3^, TLG 19.5 g, which were all below the cutoff values. The patient showed good treatment effect with SBRT, alive till the end of follow-up, whose OS and PFS time were 20.3 months and 17.6 months, respectively

**Fig. 3:**
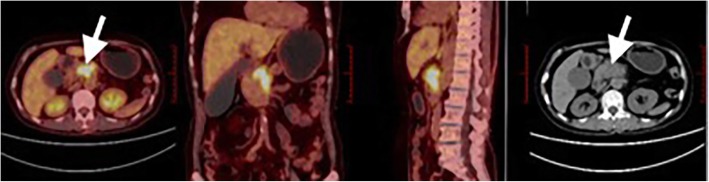
Female, 70 years old, pancreatic head cancer. ^18^F-FDG PET/CT images illustrated a lesion with increased radioactivity uptakes located in the pancreatic head. SUV_max_ 10.1 (above cutoff value), SUV_mean_ 6. (above cutoff value), MTV 9.6 cm3 (below cutoff value), TLG 59.6 g (above cutoff value). The patient had a poor outcome following SBRT, with an OS time of 11.6 months and a PFS time of 6.3 months

Typical cases of PET/CT images were presented in Figs. 2 and 3.

## Discussion

In this study, among four ^18^F-FDG PET/CT metabolic parameters, TLG showed a significant correlation with lymph node metastasis and the longest tumor diameter. Univariate analysis showed MTV, TLG, radiotherapy dose, and chemotherapy as significant prognostic factors for OS and PFS. However, lymph node metastasis and the longest diameter of the tumor were prognostic factors only for OS. In the Multivariate analysis, for LAPC patients treated with SBRT by Cyberknife, TLG, radiotherapy dose and chemotherapy were demonstrated as the independent prognostic factors for OS and PFS.

Although current research on the LAPC potential prognostic markers has reached the molecular level, such as tissue biomarkers, epigenetic markers and blood markers, including circulating tumors, few of them have been clinically recognized [12–14]. PET/CT is a technique integrating functional imaging together with anatomy imaging, which has been widely applied in the prognosis of various malignant tumors. SUV_max_, as the most commonly used semi-quantitative index in FDG PET/CT, is still controversial in LAPC prognosis assessment. Several previous studies confirmed the prognostic role of SUV_max_ in LAPC and suggested that the higher the pre-treatment SUV_max_ value of the primary lesions, the poorer the prognosis [8, 15]. Schellenberg et al.^8^ also found in a univariate analysis that the metabolic burden of the primary pancreatic tumor (similar conception to MTV) was prognostically related, but only SUV_max_ was an independent prognostic factor. However, SUV_max_ only represents the highest metabolic activity within tumor lesions and is easily affected by noise. At the same time, SUV_mean_ is calculated from the average SUV value of the entire tumor, normally varies between different operators. Moreover, SUV_mean_ can not reflect the internal metabolic characteristics of tumor. A recent study carried out by Dholakia et al. [16] found that SUV_max_ was not an independent prognostic factor for LAPC, while MTV and TLG were proved to be independent factors in LAPC prognostic value. LAPC patients with higher MTV or TLG led to poor prognosis. Similar to our study, in which SUV_max_ and SUV_mean_ were found not significantly associated with PFS or OS.

MTV refers to the volume of tumor tissue, whose FDG uptake exceeds a certain threshold (40% of SUV_max_ in this study). TLG represents the metabolic activity and metabolic volume of the tumor tissue. Compared to SUV, MTV and TLG have advantages in reflecting the tumor metabolic burden. Thus, MTV and TLG could provide a more accurate prognostic value. In recent stuides, TLG was considered as a much better indicator than MTV in prognosis evaluation [17]. It is conceptual understandable that TLG gained favour. As the product of SUVmean and MTV, TLG not only reflects the tumor volume [18], but also shows the abnormal level of metabolism inside the tumor tissue. Furthermore, TLG was proved to be strongly correlated with clinical factors, which was not showed in other metabolic parameters. Chong et al. [19] reported TLG was significantly correlated with CA19–9 level in patients with resected pancereatic cancer. Further proved the advanced oncologic prognostic value of TLG. Choi et al. [20] retrospectively analyzed 60 patients with LAPC and found the pre-treatment MTV value was an independent prognostic factor for both PFS and OS. At the same time, TLG was an independent prognostic indicator for PFS. In addition, the disease control rate (DCR) was significantly higher among LAPC patients with low MTV or TLG value. Similar results were found by our previously published work [11], in which 23 LAPC patients received chemo-SBRT combined therapy were included. And MTV was proven to be an independent prognostic factor for OS. In the current study, with a wider inclusion criterion and a larger group of research samples, both MTV and TLG are proved to be associated with PFS and OS by univariate analysis. Further tested by multivariate analysis, only TLG was an independent prognostic factor for OS and PFS. We divided TLG into 4 groups based on its quartile, and further analyzed the relationship between TLG and OS with Cox proportional hazards model. Although all the *p* values were > 0.05, the trend of higher TLG value lead to poorer prognosis could also be indicated from the analysis. The insignificant result might be related to the limited patients included in the study. Compared to our last study, the difference in analytical results may be caused by inclusion criteria. Not only LAPC patients received chemo-SBRT combined therapy were included, but also LAPC patients treated with SBRT only were enrolled in this research. Additionally, only 23 patients were analyzed in the previous study, the number was broadened to 73 patients in this study. And the difference in patient numbers may be another reason leading to the discrepant results.

As mentioned above, although a number of previous studies have discussed the prognostic value of MTV and TLG in LAPC patients, all came up with various results [16, 20]. This could be caused by different inclusion criteria, especially variable treatment options. Research carried out by Dholakia et al. [16] included 32 LAPC cases underwent SBRT, among which 27 of 32 cases were pre-treated with 1 cycle of gemcitabine-based inducing chemotherapy. In another study performed by Choi et al. [20], all 60 LAPC patients were treated with gemcitabine-based chemotherapy combined with SBRT. In our study, 73 cases of LAPC were treated with SBRT, 23 cases of which were combined with chemotherapy. Additionally, the disprepant results might also arise from unstandardized MTV delineation. The commonly used methods for MTV delineation are relative threshold method, multiple SUV absolute threshold method and gradient threshold method. A phantom study suggested that 40–50% of SUV_max_ could maximally delineate the actual tumor lesion [21]. Currently, the mainstream measurement methods of MTV include fixed SUV value, based on threshold and algorithm-based. MTV measured based on thresholds performed well in prognosis evaluation in several studies [22–24], and the method is simpler than algorithm-based. Because of tumor heterogeneity, the metabolic level of pancreatic cancer can be varied individually. When the metabolism level of pancreatic cancer is low, the fixed SUV thresholds may have a larger risk of underestimating the actual tumor volume, which will bias the results with false negative errors. Therefore, the method of MTV measurement based on threshold was applied in this study. However, if the threshold of MTV is too low, it is easy to cause an over-high background. If the threshold of MTV is too high, it may underestimate the tumor volume, and even cause partial volume effects. According to the research carried out by Dholakia et al. [25], MTV was calculated at 40% of the SUVmax value. In addition, our previous study [11] also found that MTV assessed based on a threshold of 40% was an independent prognostic factor for LAPC. For the later researches, the standardization of SUV threshold should be improved to widen the application of FDG PET/CT volume parameters in disease prognosis.

Despite the value of TLG in the prognostic assessment of SBRT treated LAPC patients, our study also showed that chemotherapy was an independent prognostic factor for PFS and OS. With chemotherapy, the survival duration of PFS and OS could be improved, which reconfirmed the role of chemotherapy in LAPC treatment [26, 27]. To date, the CRT remains to be a recommended treatment plan for LAPC patients, though the role of traditional radiotherapy was controversial. In order to offer a better treatment option, several clinical trials tried to compare the survival rates between single chemotherapy and CRT in LAPC patients, ending up with contradictory results [26, 27]. In recent years, Cyberknife therapy has been recognized by the American Society of Clinical Oncology to have a role in LAPC treatment [3]. As a newly improved SBRT equipment, the Cyberknife has been applied for LAPC treatment, and the value has been verified by several studies, as well as in our study [4, 5, 28]. It was found that the PFS and OS of LAPC patients received a higher radiotherapy dose (> 64.4 Gy) was significantly longer than those receiving a therapeutic dose (≤64.4 Gy). Similar results were found by Gurka et al. [29]. Therefore, for LAPC patients with high pre-treatment TLG, if the basic conditions and chemo-tolerance allowed, we suggested a combination treatment of chemotherapy and high radiotherapy dose improve prognosis.

There are limitations to this study. First, Considering of the defects of retrospective study, the follow-up plan was not highly standardized, which limited the robustness of PFS. In addition of censored data and selection bias. The confidence intervals in this study were not ideal, which may due to the limited number of patients limited. To get a more precised result, a randomized controlled prospective with a large sample size needs to be performed in the future work. Secondly, some images presented with diffuse uptake because of obstructive pancreatitis, it was difficult to clearly contour the tumor lesions, which might affect the measurement of MTV. Thus, ceCT and MRI images were used to help define tumor tissue. However, as ceCT and MRI and PET/CT scans were performed by different machines, there may be some deviation in the image matching. More effort will be paid and Co-scans could be performed in future work to improve the accuracy of tumor delineation. All patients underwent a ceCT scan or a MRI scan before radiotherapy, and the images were used to help differentiate pancreatic inflammatory from tumor.

## Conclusions

The ^18^F-FDG PET/CT metabolic parameters MTV and TLG were significantly associated with PFS and OS. TLG, radiotherapy dose, and chemotherapy were independent prognostic indicators of PFS and OS for LAPC patients treated with SBRT.

## Abbreviations

ASCO: American Society of Clinical Oncology
CA19–9: Carbohydrate antigen 19–9
CRT: Chemoradiotherapy
ECOG: Eastern cooperative oncology group
HR: Hazard ratio
LAPC: Locally advanced pancreatic cancer
MTV: Tumor metabolic volume
OS: Overall survival
OSEM: Ordered subset expectation maximization
PFS: Progression-free survival
PTV: Planning target volume
RADIOTHERAPY: Gross tumor volume
SBRT: Stereotactic body radiation therapy
TLG: Total lesion glycolysis

## Availability of supporting data

Supporting data could be reached by contacting the correseponding authors.
